# A New Histology-Based Prognostic Index for Aggressive T-Cell lymphoma: Preliminary Results of the “TCL Urayasu Classification”

**DOI:** 10.3390/jcm13133870

**Published:** 2024-06-30

**Authors:** Hideaki Nitta, Haruko Takizawa, Toru Mitsumori, Hiroko Iizuka-Honma, Tomonori Ochiai, Chiho Furuya, Yoshihiko Araki, Maki Fujishiro, Shigeki Tomita, Akane Hashizume, Tomohiro Sawada, Kazunori Miyake, Mitsuo Okubo, Yasunobu Sekiguchi, Miki Ando, Masaaki Noguchi

**Affiliations:** 1 Department of Hematology, Juntendo University Urayasu Hospital, 2-1-1 Tomioka, Urayasu-shi 279-0021, Japan; nitta@juntendo.ac.jp (H.N.); takizawa@juntendo.ac.jp (H.T.); t.mitsumori.db@juntendo.ac.jp (T.M.); hiiduka@juntendo.ac.jp (H.I.-H.); t.ochiai@juntendo.ac.jp (T.O.); c-furuya@juntendo.ac.jp (C.F.); 2 Division of Hematology, Juntendo University Juntendo Hospital, Tokyo 113-0033, Japan; m-ando@juntendo.ac.jp; 3 Department of Pathology and Microbiology, Division of Microbiology, Nippon University School of Medicine, Tokyo 113-8602, Japan; yaraki@juntendo.ac.jp; 4 Institute for Environmental and Gender-Specific Medicine, Juntendo University Urayasu Hospital, Chiba 279-0021, Japan; mfujishi@juntendo.ac.jp; 5 Department of Diagnostic Pathology, Juntendo University Urayasu Hospital, Chiba 279-0021, Japan; sstomita@juntendo-urayasu.jp (S.T.); akane@juntendo-urayasu.jp (A.H.); 6 Department of Clinical Laboratory, Juntendo University Urayasu Hospital, Chiba 279-0021, Japan; sawada@juntendo-urayasu.jp; 7 Department of Clinical Laboratory, Faculty of Medical Sciences, Juntendo University, Tokyo 113-8421, Japan; cpm@juntendo.ac.jp; 8 Laboratory of Blood Transfusion, Juntendo University Urayasu Hospital, Chiba 279-0021, Japan; mi-okubo@juntendo.ac.jp; 9 Hematology Clinic, Saitama Cancer Center, Saitama 362-0806, Japan; yasu_sek@saitama-pho.jp

**Keywords:** TCL, prognostic classification, GRP94, PD-L1, PD-L2, PD-1, AKR1C3, P53, GRP78, thymidine phosphorylase

## Abstract

**Background:** Aggressive mature T-cell lymphoma (TCL) is a disease that carries a poor prognosis. **Methods:** We analyzed the expression of 22 tumor cell functional proteins in 16 randomly selected patients with TCL. Immunohistochemistry was performed in paraffin-embedded tumor tissue sections to determine the protein expression statuses in tumor cells. **Results:** Glucose-regulated protein 94 (GRP94), a protein that serves as a pro-survival component under endoplasmic reticulum (ER) stress in the tumor microenvironment, was significantly associated with a shortened survival. Furthermore, significant differences were observed when GRP94 was combined with six other factors. The six factors were (1) programmed cell death-ligand 1 (PD-L1); (2) programmed cell death 1 (PD-1); (3) aldo-keto reductase family 1 member C3 (AKR1C3); (4) P53, a tumor suppressor; (5) glucose-regulated protein 78 (GRP78), an ER stress protein; and (6) thymidine phosphorylase (TP). Based on the combination of GRP94 and the six other factors expressed in the tumors, we propose a new prognostic classification system for TCL (TCL Urayasu classification). Group 1 (relatively good prognosis): GRP94-negative (*n* = 6; median OS, 88 months; *p* < 0.01); Group 2 (poor prognosis): GRP94-positive, plus expression of two of the six factors mentioned above (*n* = 5; median OS, 25 months; *p* > 0.05); and Group 3 (very poor prognosis): GRP94-positive, plus expression of at least three of the six factors mentioned above (*n* = 5; median OS, 10 months; *p* < 0.01). **Conclusions:** Thus, the TCL Urayasu prognostic classification may be a simple, useful, and innovative classification that also explains the mechanism of resistance to treatment for each functional protein. If validated in a larger number of patients, the TCL Urayasu classification will enable a targeted treatment using selected inhibitors acting on the abnormal protein found in each patient.

## 1. Introduction (A Brief Review)

Aggressive mature T-cell lymphomas (TCL) comprise peripheral T-cell lymphoma, not otherwise specified (PTCL-NOS); angioimmunoblastic T-cell lymphoma (AITL); and anaplastic large T-cell lymphoma (ALCL). TCL is commonly treated with the CHOP regimen, but these tumors are often refractory to this treatment, and the 5-year survival rate of these patients is approximately 30% [1]. While a comprehensive analysis of many genes involved in treatment resistance of malignant hematologic tumors has been performed [2], no such comprehensive analyses of functional proteins have been performed, although there have been numerous isolated reports.

In a previous study [3], we examined the expressions of 17 drug-resistant protein molecules reported previously that may be involved in the mechanism of resistance in 42 patients with newly diagnosed LBCL. Based on the results, we were able to stratify the LBCL patients into four prognostic groups: (1) very good prognosis group with negative tumor cell staining for both GRP94 and CYP3A4 (5-year OS, 100%); (2) good prognosis group with positive tumor cell staining for GRP94 and negative staining for CYP3A45- (5 year OS, about 60–80%), and either negative staining for AKR1C3 and MDR1 or MRP1 and P53; (3) poor prognosis with positive tumor staining for GRP94 and negative staining for CYP3A4, plus positive staining for at least one of AKR1C3, MDR1, MRP1, and P53 (5-year OS of about 10–20%); (4) very poor prognosis with positive tumor staining for both GRP94 and CYP3A4 (1-year OS of 0). 

In this present study, we studied in 16 patients affected with TCL the expression of various (*n* = 22) functional proteins that are considered as relevant to treatment resistance and we analyzed their ability to predict the prognosis of these patients.

The 22 types of proteins associated with treatment resistance selected by us can be classified into the following categories (1) to (5):

(1) Microenvironmental components:

Non-immune cell components in the tumor microenvironment: ER stress proteins 1.1 to 1.4 (*n* = 4):

1.1. Glucose-regulated protein 94 (GRP94) [4,5].

1.2. Glucose-regulated protein 78 (GRP78) [6].

1.3. Transforming growth factor β1 (TGFβ1) [7].

1.4. Tumor necrosis factor α1 (TNFα1) [8].

1.1 GRP94 and 1.2. GRP78 are molecules released into the extracellular space upon stress. They allow tumors to overcome various stressful conditions in the tumor microenvironment, such as hypoxia, hypoglycemia, dysregulation of homeostasis, altered cellular metabolism, and acidosis. According to references [4,5], the GRP94 epitope is located on the membrane surface of malignant cells, but not of normal cells. GRP94 is known to be strongly expressed on the surface of tumor cells in TCL, just like epidermal growth factor receptor (EGFR) in lung cancer, human epidermal receptor 2 (HER2) in breast cancer, urokinase plasminogen activator surface receptor isoform 1 (uPAR: CD87) in breast and prostate cancers, and ER-36 in breast cancer. Several undiscovered receptors are considered to interact with cell-surface GRP94. Monoclonal drugs targeting GRP94 on the tumor cell surface in TCL inhibit these interactions, to inhibit TCL progression.

1.3. TGFβ1 plays an important role in promoting tumor progression. 

1.4. TNFα1 inhibits tumor progression.

Immune cell components 1 to 3 in the tumor microenvironment (*n* = 3):

1.5. Programmed cell death 1 (PD-1) (CD279) [9].

1.6. Programmed cell death-ligand 1 (PD-L1, CD274) [9].

1.7 Programmed cell death-ligand 2 (PD-L2, CD273) [10].

In non-Hodgkin’s lymphoma, the expression of PD-1/PD-L1 on the cell surface is also important, and immune checkpoint inhibitor therapy is useful [9]. PD-L2-positive diffuse large B-cell lymphoma (DLBCL) carries a poor prognosis [11].

(2) Enzymes (2.1 to 2.5) metabolizing anticancer drugs (CHOP) (*n* = 5):

2.1. CYP3A4 [11], which degrades CHOP in PTCL patients, inactivates many anticancer drugs. It promotes inactivation of drugs inside tumors such as PTCL, causing a decrease in drug efficacy and possibly resulting in drug resistance. 2.2. CYP2B6 [12], which partially degrades CHOP, has been reported in acute leukemia. CYP2B6 is associated with an increased risk of transformation to acute lymphoblastic leukemia (ALL). As enzymes degrading CHO, 2.3. aldo-keto reductase family 1 member C3 (AKR1C3) [13] in T-cell acute lymphoblastic leukemia (T-ALL) as well as 2.4. AKR1B1 [14] and 2.5. AKR1B10 [14,15] in cancers have been reported. AKR1 is mainly present in the cytoplasm. AKR1 catalyzes the reduction of carbonyl groups to alcohols, converting them into a soluble form. AKR1 (CHO-metabolizing enzyme) also attenuates the efficacy of cyclophosphamide (C-metabolizing enzyme), adriamycin (hydroxyl doxorubicin; H-metabolizing enzyme), and oncovin (O-metabolizing enzyme; vincristine).

(3) Anticancer drugs efflux pumps 3.1 to 3.3 (*n* = 3):

3.1. Multidrug resistance protein 1 (MDR1) [2] and 3.2. multidrug resistance-associated protein 1 (MRP1) [16] in PTCL-NOS as well as 3.3. MRP4 [17] in cancers have been reported. MDR1 and MRP1 are present in the plasma membrane and are hydroxyl doxorubicin-oncovin (HO) efflux pumps. Overexpression of MDR1 and MRP1 results in tumor resistance to drugs.

(4) Other functional proteins (4.1 to 4.7) (*n* = 7):

4.1. Thymidine phosphorylase (TP) [18], which is involved in starvation resistance, angiogenesis, invasion, and metastasis, is expressed in DLBCL and other cancers. TP is said to be identical to platelet-derived endothelial cell growth factor (PDGF). The expression of TP in cancers and DLBCL is associated with a poor prognosis due to its anti-apoptotic and angiogenic effects. 4.2. p53 [19] mutations in PTCL and 4.3. MYC expression [20] in NK/T-cell lymphoma make the disease refractory to treatment. In children with acute leukemia, FLT3 gene mutations affecting the expression of 4.4. equilibrative nucleoside transporter 1 (ENT-1) [21], which mediates uptake of cylocide into leukemia cells, make the disease refractory to treatment. In malignant lymphoma and leukemia, chemotherapy including platinum-based therapy involves 4.5. Glutathione transferase (GST) [22]. Concomitant development of 4.6. fibrosis [23] and high 4.7. high Ki-67 expression [24] can also lead to a poor prognosis.

Previously reported prognostic factors (*n* = 3) IPI [25], PIT [25], and non-remission and relapse within 1 year after remission in patients receiving CHOP-like regimens [26] were used as controls.

## 2. Patients and Methods

The demographic of patients is reported in Table 1.

In this study, we enrolled 16 patients with TCL treated with CHOP as the initial remission induction therapy at our hospital during the 8-year period between 2012 and 2020, for whom the data were traceable; each of the patients provided informed consent to participate in the study. The study included 7 patients with PTCL-NOS, 6 patients with AITL, and 3 patients with ALCL. All the study subjects had received first-line CHOP therapy. The proteins that we studied were: glucose-regulated protein 94 (GRP94); glucose-regulated protein 78 (GRP78); transforming growth factor β1 (TGFβ1); tumor necrosis factor α1 (TNFα1); programmed cell death 1 (PD-1); programmed cell death-ligand 1 (PD-L1); programmed cell death-ligand 2 (PD-L2 (CD273)); enzyme metabolizing anticancer drugs CYP3A4, CYP2B6, aldo-keto reductase family 1 member C3 (AKR1C3), AKR1B1, and AKR1B10; multidrug resistance protein 1 (MDR1); multidrug resistance-associated protein 1 (MRP1); MRP4; thymidine phosphorylase (TP); p53; MYC; Ki-67; and ENT-1.

As shown in Table 1, the 16 subjects consisted of 9 males and 7 females, with a mean age of 69.3 (33 to 79) years, including 12 subjects (75%) who were aged 65 years or over. According to assessment by the Revised International Prognostic Index (R-IPI) at the first diagnosis, 9 patients (56%) were classified as poor risk. The analysis was performed using the log-rank test to compare the expressions of factors involved in the metabolism of anticancer drugs and to compare the survival rates (OS, PFS).

### 2.1. Immunohistochemistry

Immunohistopathological staining was performed using control antibodies to identify the tumor cells. Then, we determined the tumor expressions of the 22 types of treatment resistance-related proteins using the corresponding antibodies (listed in Supplementary Materials). The positivity for each immunohistochemical determination comprised the following negative and positive controls (Supplemental Table S1).

Biopsy specimens from the patients were fixed in formalin and embedded in paraffin to prepare tissue blocks, which were then sectioned and stained. The primary antibodies against the major proteins involved in anticancer drug metabolism included (1) GRP94: Proteintech (Rosemont, IL 60018, USA), clone 1H10B7 (this monoclonal antibody was generated against the N-terminal region of full-length HSP90b1); (2) CYP3A4: Sigma-Aldrich (St. Louis, MO 63103, USA), SAB1400064 (this polyclonal antibody was generated against CYP3A4); (3) AKR1C3: Proteintech, 11194-1-AP (this polyclonal antibody was generated against AKRC3); (4) MDR1 (P-glycoprotein): Proteintech, 22336-1-AP (this polyclonal antibody was generated against MDR1); (5) MRP1 (CD9): Proteintech, 60232-1-IG (this monoclonal antibody was generated against the N-terminal region of full-length MRP1); (6) TGF beta1: Proteintech, 21898-1-AP (this polyclonal antibody was generated against TGF-beta); (7) GRP78: Proteintech, 66574-1-IG (this monoclonal antibody was generated against the N-terminal region of full-length GRP78); (8) glutathione S-transferase kappa1 (GST): Proteintech, 14535-1-AP (this polyclonal antibody was generated against GST1); (9) thymidine phosphorylase: Abcam (Cambridge, UK), ab226917 (this polyclonal antibody was generated against thymidine phosphorylase); (10) MRP4 (ABCC4): SANTA CRUZ BIOTECHNOLOGY (Dallas, TX 75220, USA), SC-376262 (this monoclonal antibody was generated against the N-terminal region of full-length MRP4 (amino acid 1-280)); (11) CYP2B6: LifeSpan BioSciences, Inc. (Seattle, WA 98121, USA), LS-C352084 (this polyclonal antibody was generated against CYP2B6); (12) TNF1 alpha: Sigma-Aldrich, SAB4502982 (this polyclonal antibody was generated against TNF1 alpha); (13) PD-1; (14) PD-L1: Proteintech (Rosemont, IL, USA), 66248-1-IG, mouse IgG1 monoclonal antibody, clone 2B11D11; (15) PD-L2: Proteintech (Rosemont, IL, USA), 18251-1-AP 16, rabbit IgG polyclonal antibody; (16) P53: Cell Signaling Technology, Inc. (3 Trask Lane Danvers, MA 01923, USA), DO-7 mouse monoclonal antibody #48818; (17) c-MYC: Abcam (Kendall Sq Cambridge, MA 02139, USA), Y69 clone ab32072; (18) ENT-1 (equilibrative nucleoside transporter 1): Proteintech (Rosemont, IL, USA), 1337-1-AP rabbit IgG polyclonal antibody; (19) AKR1B1: Sigma-Aldrich (3050 Spruce Street Saint Louis, MO 63103, USA), rabbit polyclonal antibody HPA052751; (20) AKR1B10: Sigma-Aldrich (3050 Spruce Street Saint Louis, MO 63103, USA), rabbit monoclonal antibody HPA020280. After the immunostaining, two pathologists definitively determined the results of the IHC staining. The criterion for judgment of positive IHC staining was staining of more than 50% of the tumors; weakly positive abnormalities were also considered. The concordance rate between the two pathologists for the staining results was about 83%. In case of disagreement, the final diagnosis was arrived at by consensus. The surplus specimens used this time were only paraffin-embedded formalin-fixed tissues, and there were no cells preserved in a live state, making it difficult to use them for other techniques such as flow cytometry or Western blotting.

### 2.2. Statistical Analysis

To confirm the association between the OS and poor prognostic factors/factors involved in anticancer drug metabolism after the initial R-CHOP therapy, survival curves were plotted by the Kaplan–Meier method, and the factors significantly associated with the comparison of OS curves that we carried out were evaluated by the trend log-rank test.

The significance level in the statistical tests was set at 0.05 (two-tailed) and *p* < 0.05 was considered as being indicative of a statistically significant difference. Statistical analyses were performed using EZR version 2.7-1 software (Saitama Medical Center, Jichi Medical University, Saitama, Japan) [27].

Multiple comparisons were not considered because of the exploratory nature of this study.

## 3. Results

Kaplan–Meier survival curves and between-group comparisons (log-rank test).

**Figure 1:**
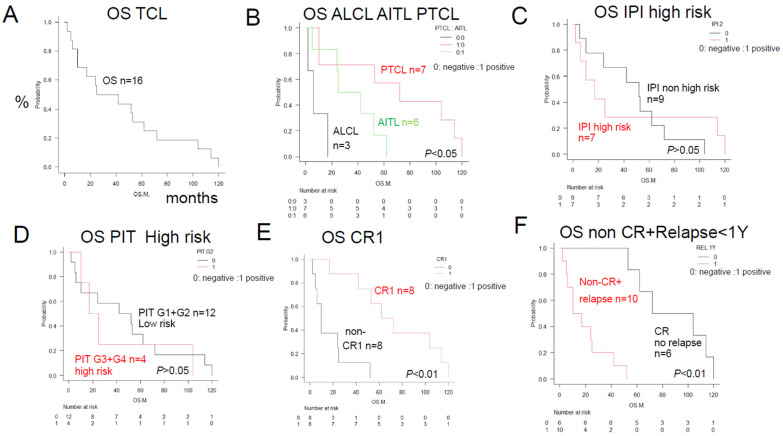
Overall survival of TCL patients with and without prognostic factors—comparison of the Kaplan–Meier survival curves and disease/existing prognostic factors between 2 groups (log-rank test)—+: positive; −: negative. (**A**) TCL overall survival (OS) (*n* = 16). The median OS after initial treatment with CHOP-like regimens was 33.5 months. (**B**) ALCL, *n* = 3; AITL, *n* = 6; PTCL NOS, *n* = 7; *p* < 0.05. The median OS values indicated a poor prognosis in the ALCL patients in this study; the OS was about 8 months in the patients with ALCL, about 34 months in the patients with AITL, and about 72 months in the patients with PTCL-NOS, (**C**) A conventional poor prognostic factor, IPI high positive, *n* = 7; negative, *n* = 9; *p* > 0.05. No significant difference was observed in the IPI among the groups. (**D**) No significant differences were observed in the distribution of the conventional poor prognostic factors in the high-risk PIT groups (Groups 2, 3, and 4; *n* = 4) as compared with the other groups (*n* = 12). No significant differences were observed in PIT among the groups (*p* > 0.05). (**E**) CR-positive and CR-negative patients (*n* = 8 in both groups). Those who showed CR had a significantly better prognosis (*n* = 8; median OS, about 72 months; *p* < 0.01). (**F**) Non-CR plus relapse within 1 year: positive, *n* = 10; negative, *n* = 6, *p* < 0.01. Patients with non-CR or relapse within 1 year had a significantly worse prognosis (*n* = 10; median OS, 13.5 months; *p* < 0.01).

As shown in Figure 1, the median OS was 33.5 months. A log-rank test and univariate analysis were performed for factors significantly affecting the OS among the conventional poor prognostic factors and factors involved in the metabolism of anticancer agents. In the subjects included in this present study, the prognosis by disease type worsened in the order of PTCL-NOS (*n* = 7; median OS, 72 months), AITL (*n* = 6; median OS, 34 months), and ALCL (*n* = 3; median OS, 8 months). No significant differences in the conventional poor prognostic factors, such as in the high-risk IPI or high-risk PIT were observed among the patients with the three disease types. However, subjects in whom complete remission (CR) was achieved showed a significantly better prognosis (*n* = 8; median OS, about 72 months; *p* < 0.01). Subjects who showed with non-CR or disease relapse within 1 year showed a significantly worse prognosis (*n* = 10; median OS, 13.5 months; *p* < 0.01).

**Figure 2:**
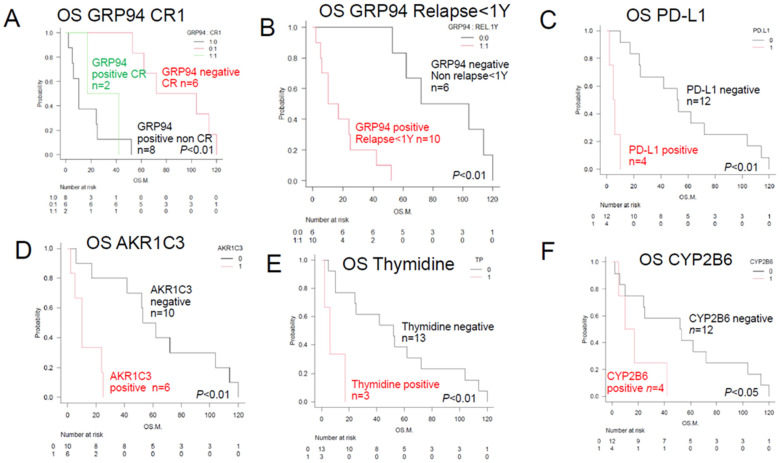
Overall survival in TCL patients with and without prognostic factors—comparison of the Kaplan–Meier survival curves and prognostic factors, positive/negative immunostaining between Group 2 and Group 3 (log-rank test). (**A**,**B**) show the results of comparisons among 3 groups, and C to F show the results of comparisons between 2 groups. (**A**) GRP94+, CR1+, *n* = 2; GRP94+, CR−, *n* = 8; GRP94−, CR+, *n* = 6; *p* < 0.01. GRP94-positive patients showed a non-CR treatment response, resulting in a poor prognosis. (**B**) GRP94-positive patients with relapse (*n* = 10), poor prognosis with a median OS of 13.5 months (*p* < 0.01). GRP94-negative patients without relapse (*n* = 6), relatively good prognosis with a median OS of 102 months (*p* < 0.01). A total of 10 patients, consisting of 6 patients who showed a non-CR treatment response after the initial therapy and 4 patients who developed relapse within 1 year, were identical to the 10 patients showing positive tumor staining for GRP94. GRP94-positive patients showed a “non-CR” treatment response after the initial therapy or developed relapse within 1 year, resulting in a poor prognosis. In the tumor microenvironment, GRP94 expression is associated with cell survival of the TCL cells and leads to a poor prognosis. In order to overcome various stressful conditions, such as altered cellular metabolism and acidosis, TCL cells survive and lead to a poor prognosis. (**C**) PD-L1-positive patients (*n* = 4, median OS, 5.5 months, *p* < 0.01); PD-L1-negative patients (*n* = 12, median OS, 50 months, *p* < 0.01). PD-L1 positivity allows TCL cells to proliferate by escaping the surveillance mechanism, which results in a poor prognosis. (**D**) AKR1C3-positive patients (*n* = 6, median OS, 10 months: *p* < 0.01); AKR1C3-negative patients (*n* = 10, median OS, 62 months; *p* < 0.01). AKR1C3 expression in TCL cells decreases the intracellular metabolism of HO of the CHOP regimen drugs, attenuating their cytotoxic activity against the TCL cells and making the disease refractory, which results in a poor prognosis. (**E**) TP-positive patients (*n* = 3, median OS, 6 months; *p* < 0.01); TP-negative patients (*n* = 13, median OS, 56 months; *p* < 0.01). TP expression is mainly linked to antiapoptotic and angiogenic activities, leading to a poor prognosis. (**F**) CYP2B6-positive patients (*n* = 4, median OS, 13.5 months; *p* < 0.05); CYP2B6-negative patients (*n* = 12, median OS, 46 months; *p* < 0.01).

As shown in Figure 2, the following five factors were identified as being significantly poor prognostic factors: (1) Glucose-related protein 94 (GRP94) expression (*n* = 10; median OS, 13.5 months; *p* < 0.01). Expression of this protein was also associated with non-CR or relapse within 1 year (*n* = 10; median OS, 13.5 months; *p* < 0.01). (2) PD-L1 expression (*n* = 4; median OS, 5.5 months; *p* < 0.01). (3) Aldo-keto reductase family 1 C3 (AKR1C3) expression (*n* = 6; median OS, 10 months; *p* < 0.01). (4) Thymidine phosphorylase (TP) expression (*n* = 3; median OS, 6 months; *p* < 0.01). (5) CYP2B6 expression (*n* = 4; median OS, 13.5 months; *p* < 0.05). The prognosis was significantly worse in the subjects with tumor expression of the aforementioned factors (1) to (5).

**Figure 3:**
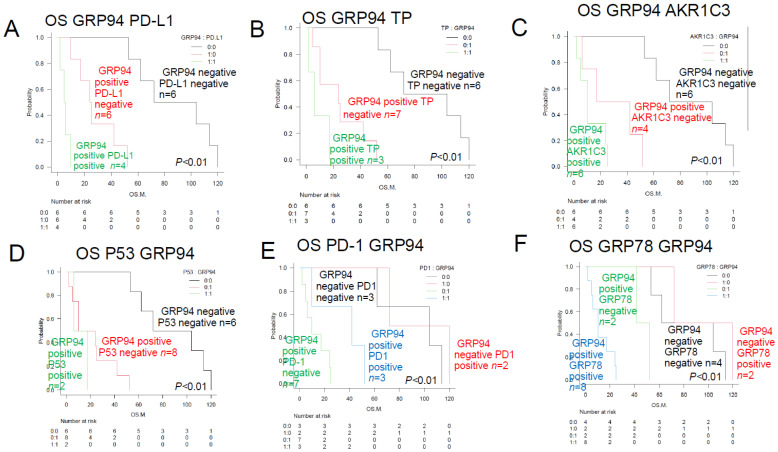
Overall survival of TCL patients with and without prognostic factors—comparison of the Kaplan–Meier survival curves and positive/negative immunostainings among 3 groups (log-rank test). (**A**) Patients showing positive tumor staining for GRP94 and PD-L1; patients showing positive tumor staining for GRP94, but negative staining for PD-L1; patients with tumors showing negative staining for both GRP94 and PD-L1. Thus, expression of GRP94 on the tumor cell surface is associated with a poor prognosis. Expression of PD-L1 on the cell surface also leads to a poor prognosis. (**B**) Patients showing positive tumor staining for GRP94 and TP; patients showing positive tumor staining for GRP94, but negative staining for TP; patients with tumors showing negative staining for both GRP94 and TP. TP expression is associated with a poor prognosis. (**C**) Patients showing positive tumor staining for GRP94 and AKR1C3; patients showing positive tumor staining for GRP94, but negative staining for AKR1C3; patients with tumors showing negative staining for both GRP94 and AKR1C3. These activities lead to a poor prognosis. (**D**) Patients showing positive tumor staining for P53 and GRP94; patients with tumors showing negative staining for P53, but positive staining for GRP94; patients with tumors showing negative staining for both P53 and GRP94. P53 mutations are associated with a poor prognosis. (**E**) Patients with tumors showing negative staining for PD-1, but positive staining for GRP94; patients showing positive tumor staining for PD-1 and GRP94; patients showing negative staining for PD-1, but positive staining for GRP94; patients showing positive tumor staining for PD-1, but negative staining for GRP94; patients showing negative staining for both PD-1 and GRP94. PD-1 signaling suppresses the growth of TCL. (**F**) Patients showing positive tumor staining for GRP78 and GRP94; patients showing positive tumor staining for GRP78, but negative staining for GRP94; patients showing negative tumor staining for both GRP78 and GRP94; patients with tumors showing negative staining for GRP78, but positive staining for GRP94. Patients showing positive tumor staining for both the ER stress proteins GRP78 and GRP94 show a poor prognosis.

The six factors (PD-L1, AKR1C3, P53, PD-1, GRP78, and TP) shown in Figure 3 also became significant when combined with GRP94 expression.

Thus, the expression of GRP94 in combination with six other factors (PD-L1, AKR1C3, P53, PD-1, GRP78, and TP) was important.

**Figure 4:**
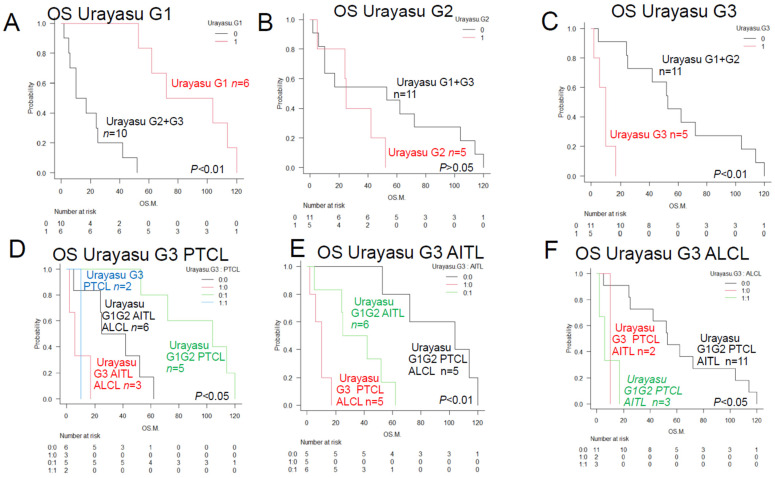
Overall survival of TCL patients with and without prognostic factors (TCL Urayasu classification)—comparison of the Kaplan–Meier survival curves among 3 groups (log-rank test). (**A**) TCL Urayasu Group 1. The median OS shown in Figure 1A almost doubled from 33.5 months, which is a relatively good prognosis for patients with TCL. For Group 2 and Group 3, the median OS was about 40% of the 33.5 months in the overall patient population, indicating a relatively poor prognosis. (**B**) TCL Urayasu Group 2 and other groups. There was no significant difference in the OS between positive groups and the negative group. The median OS in the overall patient population was about 75% of 33.5 months, indicating a relatively poor prognosis for Group 2. (**C**) TCL Urayasu Group 3 and other groups. The median OS in the overall patient population was about 30% of 33.5 months, indicating a poor prognosis for Group 3. In Group 1 and Group 2, the median OS was 53 months, being about 1.6 times that of the median OS in the overall patient population. This indicates a relatively good prognosis. (**D**) TCL Urayasu Group 3 vs. PTCL-NOS. Group 3 with PTCL-NOS and Group 3 without PTCL-NOS: Group 3 with either disease type had a poor prognosis. Group 1 or 2, with PTCL-NOS and Group 3, without PTCL-NOS. (**E**) TCL Urayasu Group 3 vs. AITL. Group 3 with AITL and Group 3 without AITL: the median OS was about 10 months in both groups. Group 1 or 2, with AITL: the median OS was about 33.5 months, which was the same as that for the OS in all patients. Patients with AITL were classified into either Group 1 or 2, and their prognosis was intermediate. Group 1 or 2, without AITL: the median OS was about 104 months. (**F**) TCL Urayasu Group 3 vs. ALCL. Group 3 with ALCL: the median OS was about 6 months. All patients with ALCL were classified into Group 3, and thus had a poor prognosis. Group 3 without ALCL: these patients had PTCL-NOS and had a median OS of about 10 months. Group 1 or 2, with ALCL and Group 1 or 2, without ALCL: the median OS was about 53 months.

Therefore, we proposed a new HPI (TLC Urayasu classification) for predicting the response to R-CHOP therapy in patients with newly diagnosed TCL. We divided the subjects into the following three groups, as shown in Figure 4: Group 1 (relatively good prognosis), patients with tumors showing negative staining for GRP94 (*n* = 8; median OS, 88 months; *p* < 0.01); Group 2 (poor prognosis), patients with tumors showing positive staining for GRP94, plus positive staining also for one or two of the six other factors (*n* = 5; median OS, 25 months; *p* > 0.05); and Group 3 (absolutely poor prognosis), patients with tumors showing positive staining for GRP94 and also positive staining for at least three of the six other factors (*n* = 5; median OS, 10 months; *p* < 0.01).

Table 2 summarizes the results of the analysis. It shows the median cumulative survival rate as assessed by the Kaplan–Meier method in the 16 TCL subjects, as well as the results of the between-group comparisons (*p*-value: log rank test). Poor prognostic factors were evaluated based on differences in the OS. Factors marked with (#), namely, GRP94, AKR1C3, CYP2B6, PD-L1, and TP, were identified as the significant poor (*p* < 0.05) prognostic factors (Figure 2). Positive tumor expression of GRP94 combined with expressions of at least one of the six other factors (PD-L1, TP, AKR1C3, P53, PD-1, and GRP78) was identified as a significant combination (*p* < 0.01) of poor prognostic factors (Figure 3).

**Figure 5:**
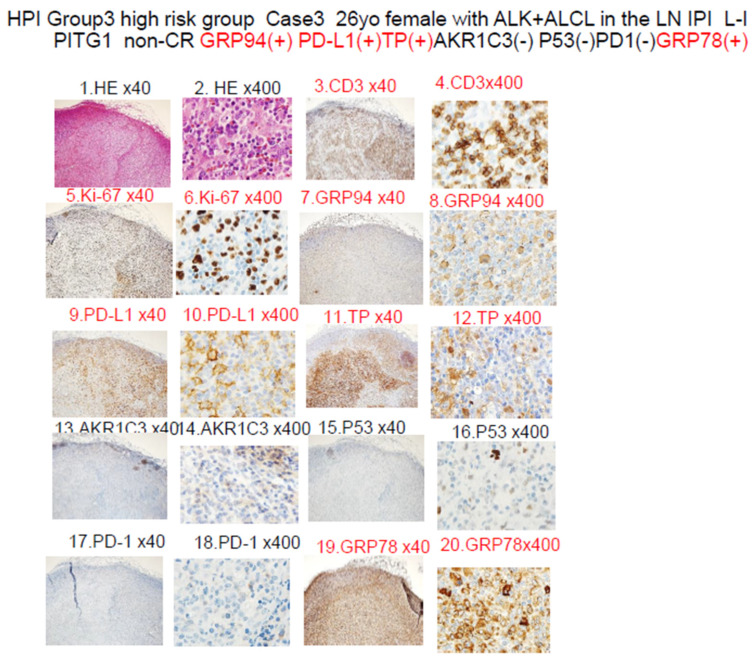
HPI (TCL Urayasu classification) Group 3 (the very poor prognosis group) Case: A 33-year-old woman diagnosed with stage IIA ALK-positive ALCL (low-intermediate IPI (IPIe), PIT-Group 1). ALK positivity is generally associated with a good prognosis. However, this patient developed resistance to both CHOP and ESHAP regimens, and died after only about 2 months of treatment. The diagnosis was ALCL. Her ALCL showed positive staining for GRP94 (shown in 8) and 3 (PD-L1, TP, and GRP78, shown in 10, 12, and 20) of the other 6 poor prognostic factors (PD-L1, TP, AKR1C3, P53, PD1, and GRP78). In the tumor microenvironment, expression of PD-L1 on the cell surface blocks the immune checkpoint molecules, allowing the tumor to grow. TP is involved in starvation resistance, angiogenesis, invasion, and metastasis. GRP78 allows the tumors to overcome various stressful conditions, such as hypoxia, hypoglycemia, dysregulation of homeostasis, altered cell metabolism, and acidosis, which results in treatment resistance.

Figure 5 shows a representative case of Group 3 (absolutely poor prognosis). The tumor showed positive staining for GRP94 and also for at least three of the six other poor prognostic factors (*n* = 5; median OS, 10 months; *p* < 0.01). The patient was a 33-year-old woman who was diagnosed as having stage IIA ALCL (low-intermediate IPI [IPIe], PIT-Group 1) (Supplemental Figures S1 and S2). After CHOP therapy and ESHAP therapy, she became resistant to treatment, and died about only 2 months after the start of treatment. Immunohistochemistry revealed positive tumor staining for GRP94, and also for three (PD-L1, TP, and GRP78) of the six other poor prognostic factors (PD-L1, TP, AKR1C3, P53, PD1, GRP78). She initially presented to us in February 2016 complaining of a left axillary mass. Biopsy of the mass revealed the diagnosis of ALK-positive ALCL (the ALK translocation partner was not NPM, and remained unknown). She showed disease progression in March 2016 after two cycles of CHOP therapy. In April 2016, after ESHAP therapy, she showed further disease progression and died (Supplemental Table S2).

## 4. Discussion

We propose a new classification, namely the HPI (TLC Urayasu classification), for predicting the response to CHOP therapy in patients with newly diagnosed TCL. As shown in Figure 4, the patients could be divided into the following three groups according to the results of IHC for the treatment resistance proteins: Group 1 (relatively good prognosis), negative tumor staining for GRP94 (*n* = 6; median OS, 88 months; *p* < 0.01); Group 2 (poor prognosis), positive tumor staining for GRP94 and also for one or two of the six other poor prognostic factors described in the text (*n* = 5; median OS, 25 months; *p* > 0.02); Group 3 (absolutely poor prognosis), positive tumor staining for GRP94 and also for at least three of the six other poor prognostic factors (*n* = 5; median OS, 10 months; *p* < 0.01) (representative case in Figure 5). As shown in Figure 4D–F, patients with ALCL (*n* = 3) and PTCL-NOS (*n* = 2) fell under Group 3 (absolutely poor prognosis). Although this present study was limited to a small sample size, the TCL Urayasu classification, which is based on the mechanism of treatment resistance of the tumors, is expected to contribute to stratified treatment, namely, the selection of the appropriate treatment depending on the disease classification.

Our findings suggest that in patients with TCL, tumor expression of GRP94 and other factors play a central role in the microenvironment, endowing tumors with the ability to adapt to adverse environmental conditions, thereby resulting in a poor prognosis. In addition to the microenvironmental component 1 (GRP94), a tumor suppressor protein (P53) and AKR1C3, which is an enzyme that inactivates CHO of CHOP, were also found to be important for the case of LBCL. Four additional microenvironmental components (PD-L1, TP, PD1, and GRP78) were found to be important for the case of TLC (Figure 3). On the other hand, for LBCL, not only the enzyme inactivating CHOP (CYP3A4), but also HO efflux pumps (MDR1 and MRP1) were found to be important prognostic factors.

Using immunohistochemistry, we examined the expressions of 17 drug-resistant protein molecules reported previously that may be involved in the mechanism of resistance to R-CHOP-like treatment in 42 patients with newly diagnosed LBCL [18].

Based on the results, we were able to stratify the LBCL patients into four prognostic groups based on the histological prognostic index (HPI): (1) very good prognosis group: 5-year OS, 100%, negative tumor cell staining for both GRP94 and CYP3A4; (2) good prognosis group: 5-year OS, about 60–80%, positive tumor cell staining for GRP94 and negative staining for CYP3A4, and either negative staining for AKR1C3 and MDR1 or MRP1 and P53; (3) poor prognosis group: 5-year OS of about 10–20%, positive tumor staining for GRP94 and negative staining for CYP3A4, plus positive staining for at least one of AKR1C3, MDR1, MRP1, and P53; (4) very poor prognosis group: 1-year OS of 0%, positive tumor staining for both GRP94 and CYP3A4. Based on the above findings, we believe that the HPI (TCL Urayasu classification) is a simple, useful, and innovative classification system for stratifying patients based on their resistance pattern to treatment, and could be expected to be applied to clinical practice in future.

**Figure 6:**
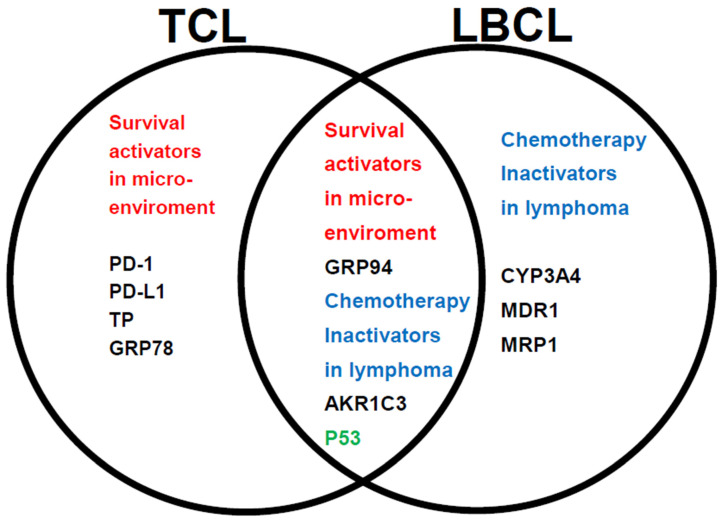
Relationships between the prognostic factors in the TCL and LBCL Urayasu classifications. The relationships between the 7 factors involved in treatment resistance in the TCL Urayasu classification and the 6 factors involved in treatment resistance in the LBCL Urayasu classification are summarized in the following set diagram Of the 7 TCL factors, the 4 factors unique to the TCL classification are the immune checkpoint molecules (PD-1 and PD-L1) serving as pro-survival components in the microenvironment, and TP and GRP78, both of which are involved in angiogenesis, invasion, and metastasis in order to overcome stressful conditions, such as hypoxia, hypoglycemia, and starvation resistance. On the other hand, the other 3 factors, which are common to both the TCL and LBCL classifications, are highly important, and consist of GRP94, AKR1C3 (an enzyme that inactivates the activity of chemotherapeutic agents by metabolizing HO), and P53 (product of a tumor suppressor gene). On the other hand, the 3 factors unique to the LBCL Urayasu classification are CYP3A4 (an enzyme that metabolizes and inactivates the activities of the CHOP regimen), MDR1, and MRP1, with both the latter being HO efflux pumps.

Figure 6 summarizes the relationship between the treatment resistance factors in the Urayasu classification for TCL and LBCL. Aggressive mature TCL (PTCL-NOS, AITL, and ALCL) tumors have at least five types of tumor pro-survival components produced and released by the tumors themselves. This indicates that TCL tumors have a better ability to adapt to the microenvironment and survive than LBCL tumors, which produce only one factor (GRP94) to survive in the microenvironment. On the contrary, according to previously reported results [28,29], LBCL tumors have a variety of at least four types of tumor chemotherapy inactivators that are produced and released by the tumors themselves. Therefore, as compared with TCL tumors, which produce only one type of enzyme, namely, AKR1C, LBCL tumors are considered as having a better ability to detoxify anticancer drugs through enzymatic degradation and promoting efflux pumping of the CHOP anticancer drugs from the tumor. These findings suggest that TCL tumors have an excellent ability to adapt to the microenvironment, and that LBCL tumors additionally have a superior ability to detoxify anticancer drugs. Furthermore, since p53 is expressed in both TCLs and LBCLs, both tumors are considered to develop mutations of TP53, a tumor suppressor gene. Furthermore, in both TCL and LBCL, GRP94, a pro-survival component, and AKR1C3, an inactivator of HO of CHOP chemotherapy, were commonly expressed in the tumor microenvironment. These results suggest that GRP94, AKR1C3, and P53, which are expressed in both TCL and LBCL, are important for the development of resistance to treatment in mature aggressive lymphomas.

In regard to the Urayasu classification for TCL shown in Figure 6, the most significant factor, namely, GRP94, and the six additional poor prognostic factors (PD-L1, TP, AKR1C3, P53, PD-1, and GRP78) are discussed below, (1) to (6), along with a review of the literature, including their potential clinical applications.

(1) GRP94, GRP78, and TP: Molecules released into the extracellular space upon stress. They allow tumors to overcome various stressful conditions in the tumor microenvironment, such as hypoxia, hypoglycemia, dysregulation of homeostasis, altered cellular metabolism, and acidosis. As shown in Figure 2 and Figure 3, GRP94 was identified as the most important factor, as in the previous study on LBCL [3]. From the aspect of treatment, pimitespib, a GRP94 inhibitor, has been used in the treatment of GISTs [30], and is also expected to be used for the treatment of adult T-cell leukemia/lymphoma (ATL) [31]. GRP94 antibody therapy for other cancers is also being investigated [5]. GRP78 is a known risk factor for high-risk ALL in children [32]. In the future, therapy targeting GRP94 [5] is expected to become available for TCL, as shown in the study on LBCL [3], which could be expected to allow long-term complete remission of the disease.

(2) PD-1/PD-L1: A cell surface immune checkpoint molecule, which is important for immune checkpoint inhibitor therapy [9]. PD-1 inhibitory therapy in classical Hodgkin lymphoma [33] and the importance of PD L1 in the diagnosis and treatment of B-cell malignant lymphoma [34] have been reported. As shown in Figure 2C, PD-L1-positive patients (*n* = 4) showed a median OS of 5.5 months (*p* < 0.01), reflecting a poor prognosis. This suggests that cell surface expression of PD-L1 in DLBCL allows the tumor to escape immune checkpoints [34].

(3) TP: It has been reported as being a poor prognostic factor in breast cancer patients [35]. TCL patients with positive tumor staining for TP also showed a poor prognosis, falling under Group 3 of the TCL Urayasu classification, and all of them (three patients) had ALCL. The median OS of TP-positive patients was 6 months (*p* < 0.01). TP inhibitors have been suggested as being useful in the treatment of glioblastomas [36].

(4) AKR1C3: AKR1C3 inhibitors enhance the efficacy of chemotherapy in AML and T-ALL [37].

(5) P53: TP53 mutations identify high-risk events in patients with peripheral T-cell lymphomas receiving CHOP-based chemotherapy [19]. Therefore, drugs tailored to specific types of p53 mutations are emerging, and p53-based immunotherapy approaches are being devised [38].

(6) PD-1: The immune checkpoint receptor PD-1 is recurrently inactivated in TCL, and is therefore said to be predictive of a poor prognosis [39]. PD1/PDL1 checkpoint inhibitors in tumor immunotherapy have been developed as a promising combination therapy [40]. However, PD-1 signaling suppresses the growth of TCL [39]. Unlike IPI and TPI, HPI (TCL Urayasu classification) is specialized for tumor resistance factors. It is an innovative classification for predicting the sensitivity to chemotherapy, even if the patient is very old and frail and has serious comorbidities. In addition, the classification is easy to use, and the results can be obtained within a short period of time.

In summary, we propose a novel prognostic classification index, namely, the new HPI (TCL Urayasu classification) for predicting the prognosis in patients with TCL; the classification requires immunohistochemistry for at least six proteins (PD-L1, AKR1C3, P53, PD-1, GRP78, and TP) in addition to GRP94 at the time of immunohistopathological diagnosis of TCL. Serology for HTLV-1 and HIV antibodies is also recommended. In the future, we expect to obtain improved treatment outcomes in patients with first-episode TCL by selecting the appropriate treatment for patients with TCL according to the HPI, including the use of the appropriate inhibitors and antibodies. It could be very valuable to include subjects from other hospitals in this study. The sample size used in this manuscript is not sufficient to make the conclusions noted in this manuscript. This paper is intended to be thought provoking. We also propose to accumulate more cases and analyze other hematologic tumors and compile the results.

## Figures and Tables

**Table 1:** Characteristics of the patients enrolled in this analysis.

	*n* = 16
Age mean (range)	69.3 (33–79)
Age > 60 years (%)	12 (75%)
Male (%)	9 (56%)
Histology	
PTCL (NOS)	7 (44%)
AITL	6 (38%)
ALCL	3 (19%)
Stage	
Stage 1–2	6 (38%)
Stage 3–4	10 (63%)
IPI	
1–2 Low risk	7 (44%)
3–4 High risk	9 (56%)
PIT	
Group 1–2 Low risk	4 (25%)
Group 3–4 High risk	12 (75%)
CHOP outcomes	
CR	8 (50%)
PD	8 (50%)
PD + relapse within 1 year	10 (63%)
Median OS (range)	38M (2–114)

Abbreviations: PTCL, peripheral T-cell lymphoma; NOS, not otherwise specified; AITL, angioimmunoblastic T-cell lymphoma; ALCL, anaplastic large T-cell lymphoma; IPI, International Prognostic Index; PIT, prognostic index for PTCL-U; CR, complete remission; PD, progressive disease; OS, overall survival.

**Table 2:** Summary of the tumor immunohistochemical findings in the patients with TCL (*n* = 16).

Category	Factors (# Significant Difference)	*n*	Median OS (Months)	*p* Value	Figure	Reference
Total	TCL	16	34		Figure 1A	
Disease	PTCL (NOS) (#)	7	72	* *p* < 0.05	Figure 1B	
	AITL (#)	6	34	* *p* < 0.05	Figure 1B	
	ALCL (#)	3	6	* *p* < 0.05	Figure 1B	
Prognostic factor	IPI High risk	7	17	*p* > 0.05	Figure 1C	
	PIT High risk	4	21	*p* > 0.05	Figure 1D	
Result	Non-CR1 (PD) (#)	8	10	** *p* < 0.01	Figure 1E	
	PD or within 1Y relapse (#)	10	14	** *p* < 0.01	Figure 1F	
ER stress proteins	GRP94 (#)	10	14	** *p* < 0.01	Figure 2B	[4,5,6]
	TGFβ1	7	25	*p* > 0.05		[9,10]
	GRP78	10	14	*p* > 0.05		[7,8]
	TNFα1	4	16	*p* > 0.05		[11]
OH metabolic enzyme	AKR1C3 (#)	6	10	** *p* < 0.01	Figure 2D	[12,13,14,15,28]
	AKR1B1	4	21	*p* > 0.05		
	AKR1B10	10	21	*p* > 0.05		
C metabolic enzyme	CYP2B6 (#)	4	14	* *p* < 0.05	Figure 2F	[18]
CHOP metabolic enzyme	CYP3A4	0				[16,17]
OH efflux pump	MDR1	1	24	*p* > 0.05		[19,20,21]
	MRP1	0				[22,23]
MTX efflux pump	MRP4	0				[24]
Immune check point molecules	PD-1	5	52	*p* > 0.06		
	PD-L1 (#)	4	6	** *p* < 0.01	Figure 2C	
	PD-L2	1	53	*p* > 0.05		
Others	TP (#)	3	6	** *p* < 0.01	Figure 2E	[27]
	p53	2	12	*p* > 0.05		
	GST	13	25	*p* > 0.05		[26]
	MYC	3	6	*p* > 0.05		
	ENT-1	16	34	*p* > 0.05		
	Fibrosis (silver stain positive)	14	39	*p* > 0.05		[28]
Significant combination						
	Non-CR1 plus GRP94+ (#)	8	10	** *p* < 0.01	Figure 2A	
	PD relapse < 1 year plus GRP94 (#)	10	10	** *p* < 0.01	Figure 2B	
	GRP94+ plus PD-L1+ (#)	4	4	** *p* < 0.01	Figure 3A	
	GRP94+ plus TP+ (#)	3	5	** *p* < 0.01	Figure 3B	[22,23,25]
	GRP94+ plus AKR1C3+ (#)	6	10	** *p* < 0.01	Figure 3C	
	GRP94+ plus P53+ (#)	2	4	** *p* < 0.01	Figure 3D	
	GRP94+ plus PD-L1+ (#)	7	10	** *p* < 0.01	Figure 3E	
	GRP94+ plus GRP78+ (#)	8	11	** *p* < 0.01	Figure 3F	
	PD relapse < 1 year plus AKR1C3+ (#)	6	10	** *p* < 0.01		
	PD relapse < 1 year plus PD-L1+ (#)	4	5	** *p* < 0.01		
	IPI High risk, GRP94+ (#)	5	10	** *p* < 0.01		
	PIT High risk, PD-L1+ (#)	4	8	** *p* < 0.01		
	TCL Urayasu G1 (#)	6	88	** *p* < 0.01	Figure 4A	
	TCL Urayasu G2	5	25	*p* > 0.05	Figure 4B	
	TCL Urayasu G3 (#)	5	10	** *p* < 0.01	Figure 4C	
	TCL Urayasu G3 PTCL (NOS)	2	10	* *p* < 0.05	Figure 4D	
	TCL Urayasu G3 AITL	0		** *p* < 0.01	Figure 4E	
	TCL Urayasu G3 ALCL	3	6	*p* < 0.05	Figure 4F	

Abbreviations: TCL, T-cell lymphoma; TP, thymidine phosphatase; MDR1, multidrug resistance protein 1; MRP1, multidrug resistance-associated protein 1; AKR1C3, aldo-keto reductase family 1 member C3; OH, oncovin + hydroxyl doxorubicin; MTX, methotrexate sodium. Factors marked with (#). The correspondence of the * mark at the significance level of the *p* value is indicated as * *p* < 0.05, ** *p* < 0.01.

## Data Availability

No additional data available.

## Supplementary Materials

The following supporting information can be downloaded at: https://www.mdpi.com/article/10.3390/jcm13133870/s1; Figure S1: Case 1: A 73-year-old woman diagnosed with stage IIIB PTCL-NOS (high-risk IPI, PIT-Group 1); Figure S2: HPI (TCL Urayasu classification) Group 2 (the poor prognosis group). Case 2: A 65-year-old woman diagnosed with stage IVB AITL (high-intermediate IPI, PIT-Group 3); Table S1: 22 antiboies list; Table S2: Summary of three representative cases shown in Figures S1, S2 and Figure 5 classified according to the histological prognostic index (TCL Urayasu classification).
