# Supplementary material for: A New Histology-Based Prognostic Index for Aggressive T-Cell lymphoma: Preliminary Results of the “TCL Urayasu Classification”
Source: J Clin Med. 2024 Jun 30;13(13):3870. doi: 10.3390/jcm13133870 (PMC11242040; doi:10.3390/jcm13133870)
Supplement: Supplementary file 1 [file jcm-13-03870-s001.zip › jcm-2981849-supplementary .pdf]

Supplementary information.

Table S1:

Table　22 antiboies list

Table	22 antibodies list				
Category	Targets	Primary antibodies	Vendor	Clone (Epitope)	Catalog number
(1) Microenvironmental components:	1. GRP94	Monoclonal antibody	Proteintech	H10B7　(N-terminal region of full-length HSP90b1)	60012-1-IG
	2. GRP78	Monoclonal antibody	Proteintech	1D6F7	66574-1-IG
	3. TGFβ1	Polyconal antibody	Proteintech	none	21898-1-AP
	4. TNFα1	Polyconal antibody	Sigma-Aldrich	none	SAB4502982
	5. PD-1	Monoclonal antibody	abcam	NAT105	ab52587
	6. PD-L1	Monoclonal antibody	Proteintech	2B11D11	66248-1-IG
	7. PD-L2	Polyconal antibody	Proteintech	none（CD273）	18251-1-AP
(2) Metabolic enzymes	1.CYP3A4	Polyconal antibody	Sigma-Aldrich	none	SAB1400064
	2.CYP2B6	Polyconal antibody	LifeSpan BioSciences, Inc	none	LS-C352084
	3.AKR1C3	Polyconal antibody	Proteintech	none	11194-1-AP
	4.AKR1B1	Polyconal antibody	Sigma-Aldrich	none	HPA052751
	5.AKR1B10	Polyconal antibody	Sigma-Aldrich	none	HPA020280
(3) Drugs efflux pumps	1.MDR1	Polyconal antibody	Proteintech	none	22336-1-AP
	2.MRP1	Monoclonal antibody	Proteintech	4H7B9 (CD9)	60232-1-IG
	3.MRP4	Monoclonal antibody	SANTA CRUZ BIOTECHNOLOGY	F-6	SC-376262
(4)Functional proteins	1. TP	Polyconal antibody	abcam	none	ab226917
	2. P53	Monoclonal antibody	GeneTex	DO-7	GTX75255
	3. c-MYC	Monoclonal antibody	abcam	Y69	ab32072
	4. ENT-1	Polyconal antibody	Proteintech	none	1337-1-AP
	5. GSTk1	Polyconal antibody	Proteintech	none	14535-1-AP

6. Ki-67	Monoclonal antibody	Dianova GmbH	MIB-1	DIA-670-P1
7. CD109	Polyconal antibody	Sigma-Aldrich		HPA015723

Table 22 antibodies list

Category	Targets	Primary antibodies	Vendor	Clone (Epitope)
(1) Microenvironmental components:	1. GRP94	Monoclonal antibody	Proteintech	H10B7 (N-terminal region of full-length HSP90b1)
	2. GRP78	Monoclonal antibody	Proteintech	1D6F7
	3. TGFβ1	Polyconal antibody	Proteintech	none
	4. TNFα1	Polyconal antibody	Sigma-Aldrich	none
	5. PD-1	Monoclonal antibody	abcam	NAT105
	6. PD-L1	Monoclonal antibody	Proteintech	2B11D11
	7. PD-L2	Polyconal antibody	Proteintech	none (CD273)
(2) Metabolic enzymes	1.CYP3A4	Polyconal antibody	Sigma-Aldrich	none
	2.CYP2B6	Polyconal antibody	LifeSpan BioSciences, Inc	none
	3.AKR1C3	Polyconal antibody	Proteintech	none
	4.AKR1B1	Polyconal antibody	Sigma-Aldrich	none
	5.AKR1B10	Polyconal antibody	Sigma-Aldrich	none
(3) Drugs efflux pumps	1.MDR1	Polyconal antibody	Proteintech	none
	2.MRP1	Monoclonal antibody	Proteintech	4H7B9 (CD9)
	3.MRP4	Monoclonal antibody	SANTA CRUZ BIOTECHNOLOGY	F-6
(4)Functional proteins	1. TP	Polyconal antibody	abcam	none
	2. P53	Monoclonal antibody	GeneTex	DO-7
	3. c-MYC	Monoclonal antibody	abcam	Y69
	4. ENT-1	Polyconal antibody	Proteintech	none
	5. GSTk1	Polyconal antibody	Proteintech	none
	6. Ki-67	Monoclonal antibody	Dianova GmbH	MIB-1
	7. CD109	Polyconal antibody	Sigma-Aldrich	

Presentation of three a representative cases, one each from Group 1, Group 2, and Group 3 classified according to the HPI (TCL Urayasu Classification), focusing on the findings of immunohistochemistry.

Figures 5A, 5B, and 5C show a representative case each from of Group 1, Group 2, and Group 3 classified according to the HPI (TCL Urayasu Classification), along with their respective immunohistochemical staining images.

The details are described below. Table 3 summarizes the results.

Table S2

Table 3. Summary of three representative cases shown in Figure 5A,5B and 5(C) classified according to the histological prognostic index (TCL Urayasu classification).

Figure 5	Case	Age	Disease	IPI	PIT	TCL-Urayasu	Main factor	Additional 6 factors						Treatment	Outcome	OS
		Sex				Figure 4A-F	GRP94	PD-L1	TP	ANR1C3	P53	PD-1	GRP78			months
A	1	73F	PTCL	High	G1	Group 1	(-)	(-)	(-)	(-)	(-)	(-)	(-)	CHOP x 6	CR1	114
			(NOS)			Relatively good prognosis									Alive	
B	2	65F	AITL	HI	G3	Group 2	(+)	(-)	(-)	(+)	(-)	(-)	(+)	CHOP x 3	non-CR	25
						Poor prognosis								5 regimens	Dead	
C	3	4F	ALCL	LI	G3	Group 3	(+)	(+)	(+)	(-)	(-)	(-)	(+)	CHOP x 2	PD	2
						Very poor prognosis								ESHAP x 1	Dead	

Notes: IPI, International prognostic index; PIT, Prognostic index for PTCL-U; TCL Urayasu, T-cell lymphoma Urayasu classification; G1, Group 1; G2, Group 2; ESHAP, etoposide, methylprednisolone (solumedrol), high-dose cytarabine (ara-C), and cisplatin (platinum).

Figure 5A shows a representative case (Case 1) of Group 1 (relatively good prognosis); the tumor was negative for GRP94 (n = 6; median OS, 88 months; $p < 0.01$). The patient was a 73-year-old woman who was diagnosed as having stage IIIB PTCL-NOS (high-risk IPI, PIT-Group 1). She achieved long-sustained complete remission after 6 courses of CHOP therapy. She survived for 114 months without relapse. Immunohistochemical staining showed a negative staining result of the tumor for GRP94; the tumor also showed negative staining results for all of the 6 other poor prognostic factors (PD-L1, TP, AKR1C3, P53, PD1, and GRP78). She initially presented in June 2014 with fever and pancytopenia. CT revealed multiple enlarged lymph nodes in the left supraclavicular fossa, left axilla, mediastinum, abdominal periaortic area, and abdominal cavity, along with pleural effusion and ascites. Therefore, under the suspicion of malignant lymphoma, biopsy of an enlarged left supraclavicular fossa lymph node was performed, which confirmed the diagnosis of PTCL-NOS.

Figure 5B shows a representative case of Group 2 (poor prognosis); The tumor showed positive staining for GRP94 and also for one or two of the 6 other factors (n = 5; median OS, 25 months; $p > 0.05$). The patient was a 65-year-old woman who was diagnosed as having stage IVB AITL (high-intermediate risk IPI, PIT-Group 3). She developed resistance to CHOP and also to 5 types of salvage

treatment regimens, and died 25 months after the diagnosis. Immunohistochemistry revealed positive staining of the tumor for GRP94. Of the 6 other poor prognostic factors (PD-L1, TP, AKR1C3, P53, PD1, and GRP78), her tumor showed positive staining for AKR1C3 and GRP78. She was diagnosed as having AITL by left inguinal lymph node biopsy in July 2017. She showed partial remission after 3 cycles of CHOP therapy. However, in October 2017, her condition worsened again, and she presented with worsened splenomegaly. In November 2017, she was started on forodesin, a purine nucleoside phosphorylase (PNP) inhibitor drug. Positron emission tomography with computer tomography (PET/CT) indicated a relapse of the disease, showing multiple nodules and granular shadows in the lung fields. In February 2018, after 4 cycles of pralatrexate, she was diagnosed as having disease progression, and concomitantly developed Epstein-Barr virus (EBV)-related lymphoproliferative disease. Further disease progression was observed in January 2019 after CHOP therapy, in February 2019 after 5 cycles of romidepsin, and in July 2019 after treatment with mogamulizumab. She died in August 2019.

Figure5C means Figure 5 in the text.

Figure S1

Figure 5A. Case 1: A 73-year-old woman diagnosed with stage IIIB PTCL-NOS (high-risk IPI, PIT-Group 1).

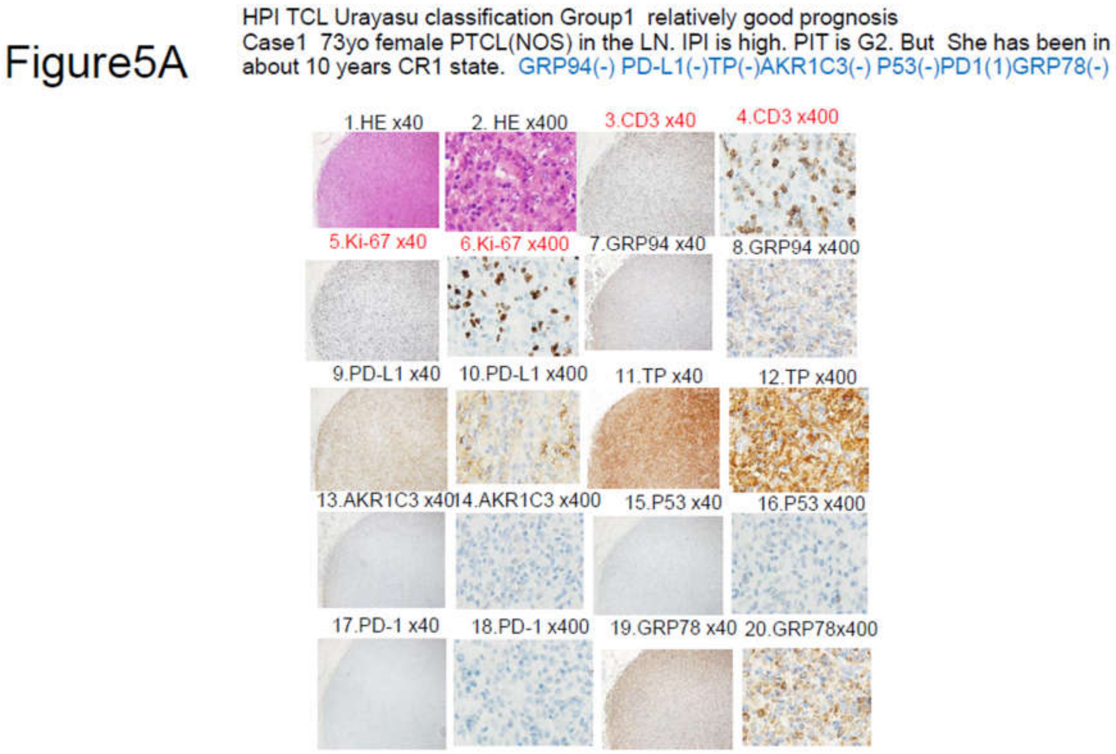

The diagnosis of PTCL-NOS was made based on the presence of medium-to-large atypical cells that were detected by HE staining and showed positive staining for CD3 Her PTCL showed a high KI-67 index and showed negative staining for GRP94. She tested negative for any other tumor cells. Therefore, she was classified into TCL Urayasu Classification Group 1 (relatively good prognosis). In fact, she has remained in complete remission for a long period of time after 6 cycles of CHOP therapy, surviving for 114 months without relapse. The absence of treatment resistance factors may account for her good prognosis.

Figure S2

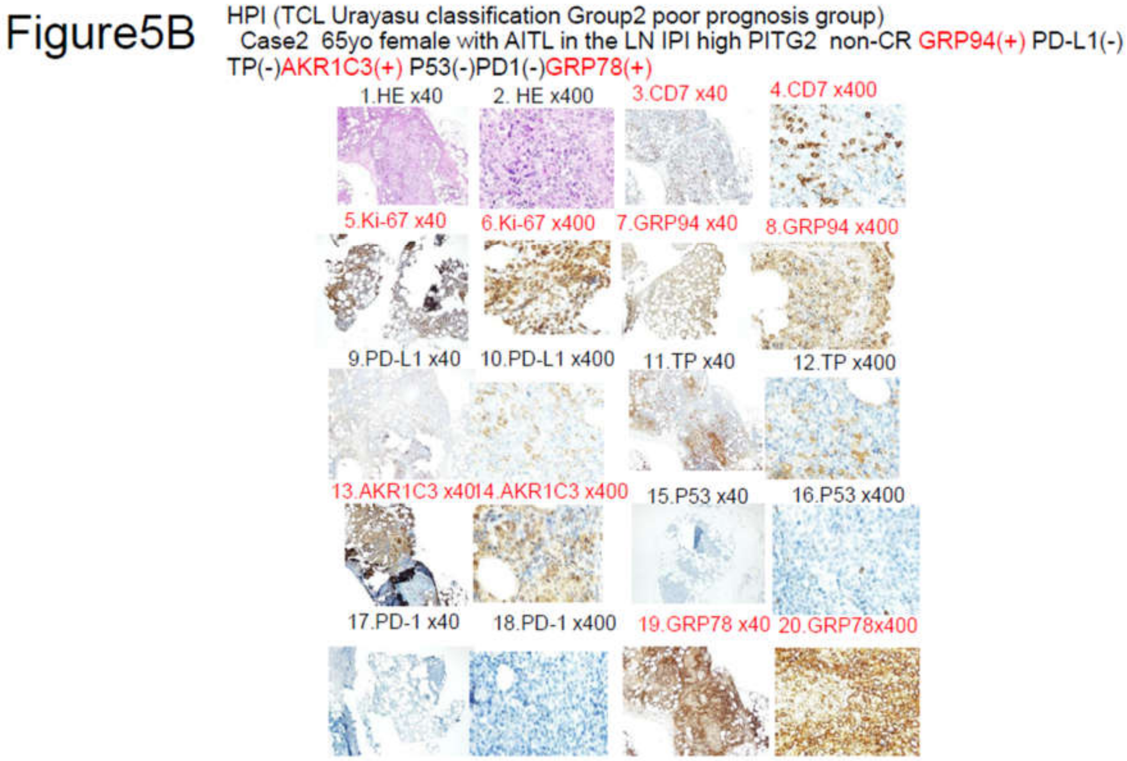

Figure 5B. HPI (TCL Urayasu classification) Group 2 (the poor prognosis group). Case 2: A 65-year-old woman diagnosed with stage IVB AITL (high-intermediate IPI, PIT-Group 3)

that showed positive immunohistochemical staining for CD7 and a highly positive Ki-67 index. She did not achieve CR after CHOP therapy, developed resistance to CHOP treatment as well as to 5 types of salvage treatment regimens, and died after surviving for 25 months after the diagnosis. Her tumor showed positive staining for GRP94, AKR1C3, and AKR1C3. The failure to achieve CR could be attributable to a reduced treatment effect due to metabolism of HO chemotherapeutic agents by intracellular AKR1C3. In addition, GRP94 and GRP78 are both ER stress proteins expressed on the cell surface that allow tumors to overcome various stressful conditions in the tumor microenvironment, such as hypoxia, hypoglycemia, dysregulation of homeostasis, altered cell metabolism, and acidosis, which results in resistance to various treatment regimens.
